# Constructing *Escherichia coli* co-display systems for biodegradation of polyethylene terephthalate

**DOI:** 10.1186/s40643-023-00711-x

**Published:** 2023-12-08

**Authors:** Jiayu Hu, Yijun Chen

**Affiliations:** Laboratory of Chemical Biology, School of Life Science and Technology, China Pharmaceutical University, Nanjing, 211198 Jiangsu People’s Republic of China

**Keywords:** PET biodegradation, *Escherichia coli*, Surface display, Adhesion

## Abstract

**Background:**

The accumulation of fast-growing polyethylene terephthalate (PET) wastes has posed numerous threats to the environments and human health. Enzymatic degradation of PET is a promising approach for PET waste treatment. Currently, the efficiency of various PET biodegradation systems requires further improvements.

**Results:**

In this work, we engineered whole cell systems with co-display of strong adhesive proteins and the most active PETase for PET biodegradation in *E. coli* cells. Adhesive proteins of cp52k and mfp-3 and Fast-PETase were simultaneously displayed on the surfaces of *E. coli* cells, and the resulting cells displaying mfp-3 showed 50% increase of adhesion ability compared to those without adhesive proteins. Consequently, the degradation rate of *E. coli* cells co-displaying mfp-3 and Fast-PETase for amorphous PET exceeded 15% within 24 h, exhibiting fast and thorough PET degradation.

**Conclusions:**

Through the engineering of co-display systems in *E. coli* cells, PET degradation efficiency was significantly increased compared to *E. coli* cells with sole display of Fast-PETase and free enzyme. This feasible *E. coli* co-display system could be served as a convenient tool for extending the treatment options for PET biodegradation.

**Graphical Abstract:**

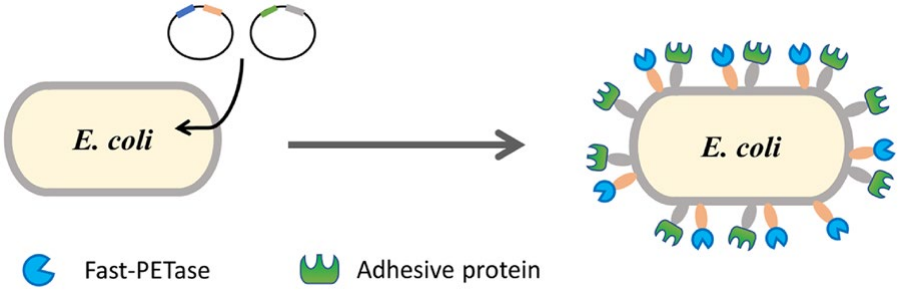

**Supplementary Information:**

The online version contains supplementary material available at 10.1186/s40643-023-00711-x.

## Introduction

**Fig. 1:**
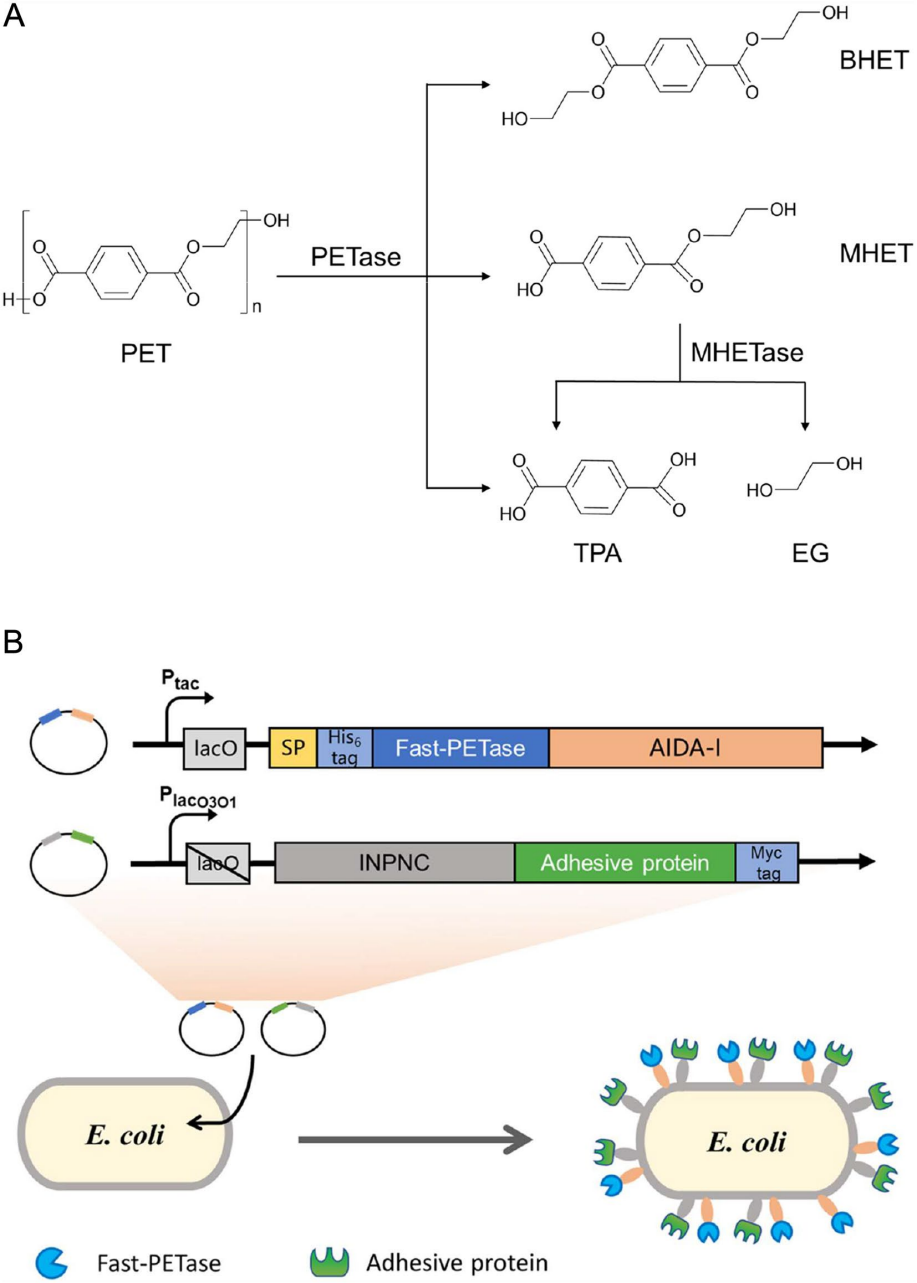
Construction of *E. coli* co-display system for PET biodegradation. **A** PET hydrolyzed into BHET, MHET and TPA by PETase, and MHET is further hydrolysed by MHETase to TPA and EG. **B** An illustration of the co-display systems and the key elements of two plasmids in the co-display system in this study. SP, signal peptide; lacO, lac operator

Polyethylene terephthalate (PET) is an aromatic polyester plastic with the largest production and consumption in the world. The extensive use of PET has generated a huge amount of PET wastes. Although PET wastes could be treated by chemical methods, the requirement of valuable energy and costly catalyst and the production of environmentally harmful effluent has been a major obstacle for applications. By contrast, biodegradation is a green and efficient way for PET waste treatment. Up to date, several PET hydrolases have been reported. Among them, IsPETase (Yoshida et al. 2016), discovered in *Ideonella sakiensis* in 2016, has been regarded as the most promising enzyme. IsPETase could efficiently hydrolyse the ester bond of PET under ambient temperatures to generate bis(2-hydroxyethyl) terephthalate (BHET), mono(2-hydroxyethyl) terephthalate (MHET) and terephthalic acid (TPA), and the most abundant hydrolytic product MHET could be further broken down into TPA and ethylene glycol by another enzyme, MHETase, derived from the same bacterium (Fig. 1A). To overcome the limitations of using free enzymes, such as instability and time-consuming purification process, various whole cell systems have been constructed for PET biodegradation using the PETases from different sources (Chen et al. 2022, 2020; Gercke et al. 2021; Jia et al. 2022; Zhu et al. 2022). However, the efficiency of PET biodegradation by these systems is still lower than realistic applications.

Because PET is an insoluble polymer with a hydrophobic and smooth surface, it is necessary for PETases to bind with the surface of PET before hydrolysis taking place based on the surface erosion process for enzymatic PET hydrolysis (Kawai et al. 2019). Yet, it remains as a difficult task because all available PETases lack specific substrate binding domains (Joo et al. 2018). Consequently, the substrate binding process is a rate-limiting step in PET biodegradation (Katyal and Montclare 2020).

Previously, carbohydrate-binding proteins (Dai et al. 2021; Xue et al. 2021) and fungus-derived hydrophobins (Puspitasari and Lee 2021; Ribitsch et al. 2015) have been fused with PET hydrolases to elevate their catalytic performances. Unfortunately, only limited increases of the efficiency on PET degradation were observed (usually 0.2-0.6-fold of improvement). Recently, a co-display system with a hydrophobin and a variant of PETase on the cell surfaces of yeasts was reported for PET degradation (Chen et al. 2022). Despite the remarkable increase of the efficiency for degrading high-crystallinity (45%) PET compared to purified free PETase at low concentrations, this yeast co-display system is still far from ideal for industrial application, mainly because it takes 10 days to reach ~ 10.9% degradation. In addition, in this yeast co-display system, the relatively weaker adhesive ability of the fungus-derived hydrophobin and slower hydrolytic rate of PETase variant (S160A) might also limit its efficiency on PET degradation.

To address above-mentioned shortcomings for further improvement, we sought to introduce PET adhesion modules to *Escherichia coli* systems for facilitating the substrate binding process with stronger adhesive proteins along with the use of the best variant of IsPETase for PET biodegradation.

To accomplish strong adhesions, the selection of adhesive proteins is critically important. Two adhesive proteins were utilized and compared in this study according to the properties and previous reports. The first one is cp52k (GenBank ATB53756.1) from stalked barnacle *Pollicipes pollicipes* (Rocha et al. 2019), a hydrophobic protein belonging to barnacle cement proteins (CP) and vital for the underwater adhesion of stalked barnacles. Because cp52k contains over 50% hydrophobic amino acids, this characteristic makes it possible to form strong hydrophobic reactions with PET surfaces. The other is mfp-3 (GenBank BAB16314.1), a type of mussel adhesive protein with only 46 amino acids. This protein is known to form strong π-π and cation-π interactions with polystyrene (PS) and polymethylmethacrylate (PMMA) (Lu et al. 2013), which has been extensively used as an adhesive for various purposes (Yang et al. 2013). To achieve effective surface display of these adhesive proteins, ice-nucleation protein (INP), an outer membrane protein of *Pseudomonas syringae* (Wolber 1993), was employed to facilitate the process. INP is composed of a N-terminal domain, a C-terminal domain and a highly repetitive central domain. For optimal anchoring behaviors, a truncated form of INP containing the N- and C-terminal portions (INPNC) was previously used as an anchor protein to effectively display target proteins on cell surfaces (Jung et al. 1998).

The reasons of choosing *E. coli* as a chassis were based on its inherited advantages, including shorter doubling time, higher clonal homogeneity, clearer genetic background for manipulations and the ease and lower costs for high-density cultivation (Lee 1996; Pontrelli et al. 2018; Schwarzhans et al. 2017). Moreover, *E. coli* is the most frequently used bacterial host for surface display with multiple carrier proteins to choose and utilize (van Bloois et al. 2011). As shown in Fig. 1B, dual surface display of an adhesive protein for PET binding and a PETase for the hydrolysis of PET would produce a synergistic effect on PET degradation, which could result in a significant enhancement of degradation efficiency.

## Materials and methods

### Strains and plasmid constructions

*E. coli* DH5α was used as the host for cloning, and *E. coli* BL21 (DE3) was used as the host for protein expression and the construction of whole cell biocatalysts. The details of all strains, plasmids are summarized in Additional file 1: Table S1 and S2.

Recombinant plasmids were constructed using Vazyme ClonExpress MultiS One Step Cloning Kit C113 (Nanjing, China). To display the adhesive proteins (cp52k and mfp-3) on the surfaces of *E. coli* cells, the proteins were individually fused to INPNC (Jung et al. 1998), yielding fusion proteins of INPNC-cp52k and INPNC-mfp with a myc tag at their C-termini. A moderate lacO_3_O_1_ promoter combined with a lac operator was used to control the inducible expression of the fusion proteins. To display Fast-PETase (Lu et al. 2022) on the surfaces of *E. coli* cells, a pelB signal peptide (MKYLLPTAAAGLLLLAAQPTMA) followed with a 6 $\times$ His-tag was put at the N-terminus of Fast-PETase, and the linker and β-barrel domains of yfaL (residues 743-1250; Swiss-Prot Entry P45508) were fused to the C-terminus of Fast-PETase, generating a fusion protein of AIDA-Fast-PETase. A moderately strong tac promoter combined with a lac operator was used to control inducible expression of this fusion protein. The key elements for the construction of plasmids are shown in Fig. 1B.

### Cultivation conditions and protein expression

*E. coli* DH5α and *E. coli* BL21(DE3) cells carrying various plasmids were cultured in Luria–Bertani broth (LB) medium (0.5% yeast extract, 1% tryptone and 1% NaCl) with corresponding antibiotics (50 µg/mL) at 37 °C. For the induction of protein expression, 1 mL of overnight cell culture was inoculated into 50 mL LB medium with corresponding antibiotics (50 µg/mL). Protein expression was induced by 0.1 mM IPTG addition when OD_600_ reached 1.0–1.2, and then continued at 25 °C for 20 h.

### Protein analysis

Cell membrane proteins were extracted using the membrane protein extraction kit (BestBio, Shanghai, China) for Western blotting analysis. For immunoprecipitation, cell membrane proteins extracted from 25 mg wet weight of *E. coli* cells were incubated with 70 μL A/G-agarose (Proteintech, #PR40025) and 2.5 μL primary anti-His mouse IgG antibody (Proteintech, #66005-1-Ig) in 1 mL phosphate buffer solution (PBS) at 4 °C overnight. Then, the beads were washed with PBS, and the immunoprecipitates were loaded for Western blotting analysis.

To establish a grayscale-protein concentration standard curve, Fast-PETase with a N-terminal 6 $\times$ His-tag was expressed in *E. coli* and purified by Ni-affinity chromatography. The purified His-tagged Fast-PETase with different concentrations was loaded for Western blotting analysis to establish the grayscale-protein concentration standard curve. To obtain displayed Fast-PETase protein, the membrane fraction of *E. coli* cells displaying Fast-PETase was extracted and immunoprecipitated (IP) with the antibody against His-tag for purifying and enriching Fast-PETase in the membrane fractions. Then, the IP samples were analyzed by Western blotting to quantify the exact amount of Fast-PETase displayed on the cell surfaces.

### Immunofluorescent microscopy

Cells were harvested and washed with PBS, and resuspended in PBS with 1% BSA at 30 °C for 1 h. After blocking, samples were incubated with anti-His mouse IgG antibody (Proteintech, #66005-1-Ig, 1:1000 dilution) and anti-myc rabbit IgG antibody (Proteintech, #60003-2-Ig, 1:1000 dilution) at 4 °C overnight. After washing with PBS, the cells were resuspended in PBS and incubated with goat anti-rabbit IgG H&L (Alexa Fluor® 647) (Abcam, #ab150079, 1:500 dilution) and goat anti-mouse IgG H&L (Alexa Fluor® 488) (Abcam, #ab150113, 1:500 dilution). Finally, cells were washed with PBS for immunofluorescent microscopy.

### PET adhesion assay

PET sheets were cut from commercial PET bottles with the size of 0.5 mm × 0.4 mm. The PET sheets were soaked with 75% ethanol and dried and then placed into a sterile 24-well plate incubated with 1 mL of *E. coli* cells at room temperature overnight.

For crystal violet (CV) assay, culture broths were discarded, and 1 mL sterile water was added into the wells and washed three times to remove weakly absorbed cells. Then, the wells were incubated with crystal violet. After washing with sterile water twice, the cells adhering to the PET sheets were examined under a microscope with oil immersion lens.

For colony-forming unit (CFU) assay, PET sheets were gently rinsed with sterile water, and the sheets were put into a tube containing 2 mL sterile water. The tube was vigorously shaken to release the attached cells. To remove residual *E. coli* cells on PET sheets, the sheets were placed in LB media and shaken at 37 ℃ for 24 h. The cell suspension was tenfold serially diluted with sterile water. Then, 50 μL of diluted sample was spread onto LB agar plates containing kanamycin and incubated at 37 °C for 12 h. The colony numbers on the plates were counted for the calculation of cell density (CFU/cm^2^) on the PET sheets.

### Whole cell biodegradation of PET

To generate amorphous PET, commercial PET bottles were cut into small pieces and dissolved in 1,1,1,3,3,3-hexafluoro-2-propanol with a final concentration of 50 mg/mL. In each tube, 4 μL of this solution was added for the precipitation of amorphous PET (Meng et al. 2021). Every 8 mg wet weight of *E. coli* cells were resuspended with 50 mM Glycine–NaOH buffer (pH 9.0) to 200 μL in 2 mL tube. Amorphous PET (0.2 mg) was added into the tube. For purified free PETase, an equal amount of Fast-PETase displayed in 8 mg wet weight of the engineered strain was added to the same system. Then, the tube was shaken with a thermomixer at 30 °C for 24 h.

### HPLC analysis of PET degradation products

Reaction tubes from above PET biodegradation were centrifuged to remove *E. coli* cells. The supernatants (approximately 200 μL) were mixed with 100 μL methanol with shaking. Then, the tubes were centrifuged again, and the supernatants were used as the samples for HPLC analysis.

HPLC was performed on a 1260 Infinity II LC System (Agilent, Santa Clara, USA) equipped with a Phenomenex Gemini C18 column (dimensions: 250 × 4.6 mm, pore size: 110 Å, particle size: 5 µm). Mobile phase: water containing 0.1% formic acid (A), acetonitrile containing 0.1% formic acid (B); elution gradient: 0–20 min: 10% B—50% B, 20–25 min: 50% B—90% B, 25–28 min: 90% B—10% B, 28–30 min: 10% B; column temperature: 40℃; flow rate: 1 mL/min; wavelength: 240 nm; injection volume: 20 µL. Different concentrations of MHET, TPA and BHET were analyzed to generate standard curves based on peak areas.

### Calculation of PET degradation rate

The degradation rate (%) of PET was calculated according to following equation (Gercke et al. 2021):

$$\frac{\left[n\left(TPA\right)+n\left(MHET\right)+n\left(BHET\right)\right]\times \left[M\left(MHET\right)-M({H}_{2}O)\right]}{m(PET)}\times 100=Degradation(\%)$$

$n\left(TPA\right)$, $n(MHET)$ and $n\left(BHET\right)$: molar amount of degradation products of TPA, MHET and BHET, respectively; $M\left(MHET\right)$ and $M({H}_{2}O)$: molecular weight of MHET and H_2_O; $m(PET)$: mass of PET substrate.

## Results

### Sole-display of adhesive proteins on the surfaces of *E. coli* cells

To achieve desired adhesion with PET, appropriate adhesive proteins are a vital factor for surface display. Given the strong underwater adhesion ability of certain marine organisms, we expected that those marine organism-derived proteins would exert stronger adhesion for PET. Subsequently, two marine organism-derived adhesive proteins, cp52k and mfp-3, were selected for evaluation.

**Fig. 2:**
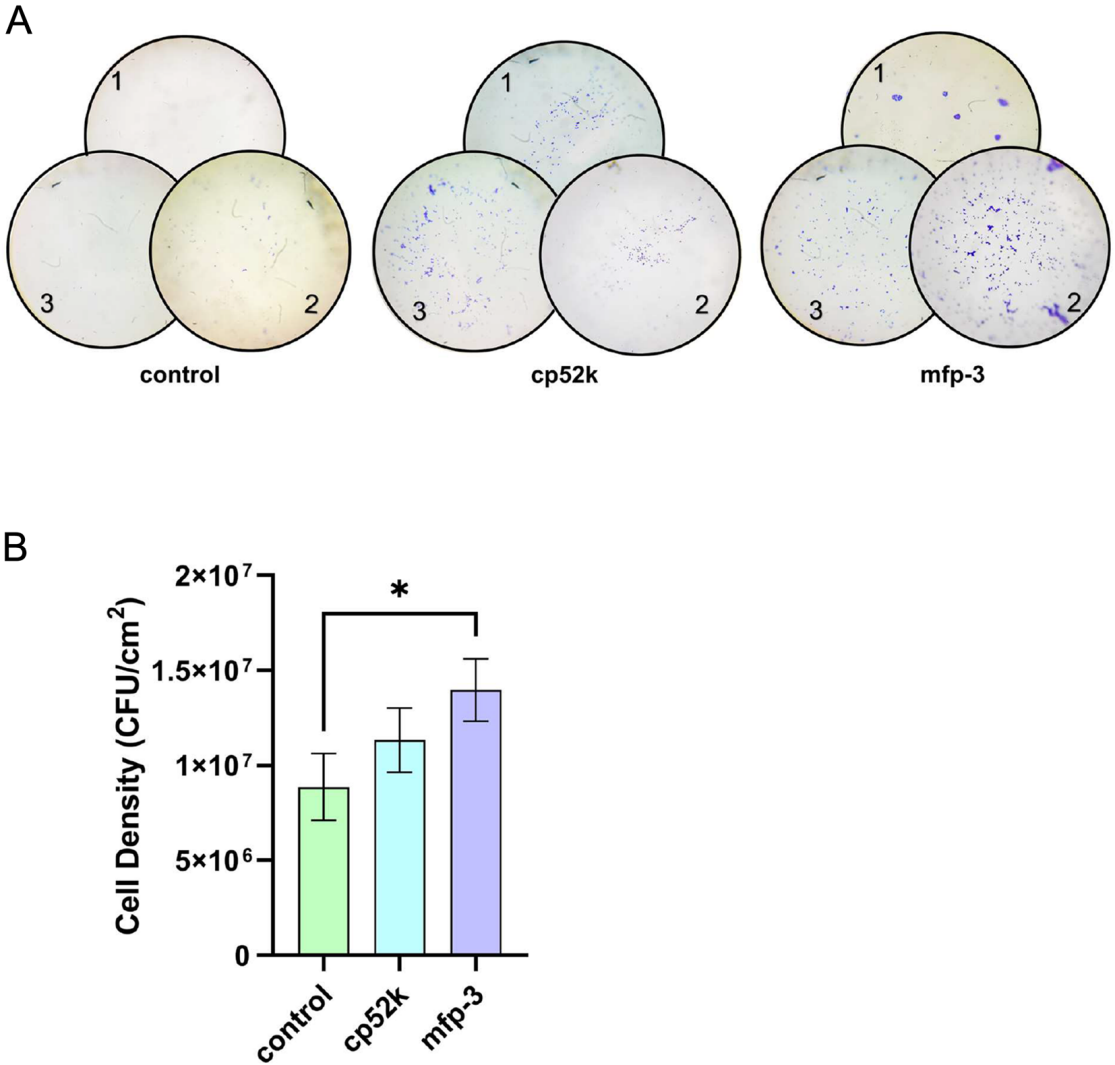
The adhesive performance of *E. coli* cells to PET. **A** Microscopic images of *E. coli* cells adhering with PET (100 ×). Three independent experiments are shown with numbers. Control represents *E. coli* cells with empty vector. **B** Cell density (CFU/cm^2^) of *E. coli* cells adhering with PET. All experiments were conducted in triplicate. The values represent mean ± SD, and the asterisk denotes statistically significant difference (*p* < 0.05, unpaired t-test)

After successful display of INPNC-cp52k and INPNC-mfp in *E. coli* BL21(DE3) using INPNC as an anchor protein, the adhesion ability of the engineered *E. coli* cells with PET was examined. As shown in Fig. 2A, the number of bacterial cells displaying either cp52k or mfp-3 on PET sheets was more than that of the cells without adhesive proteins. Meanwhile, CFU was counted to quantify the cells adhering to PET sheets (Fig. 2B). Under such conditions, the cell numbers for control, cp52k and mfp-3 were 8.9 $\times$ 10^6^, 1.1 $\times$ 10^7^ and 1.4 $\times$ 10^7^ per cm^2^. Comparted to *E. coli* cells without adhesive proteins, the display of mfp-3 showed a 50% increase of adhering cell number.

### Sole-display of Fast-PETase on the surfaces of *E. coli* cells

**Fig. 3:**
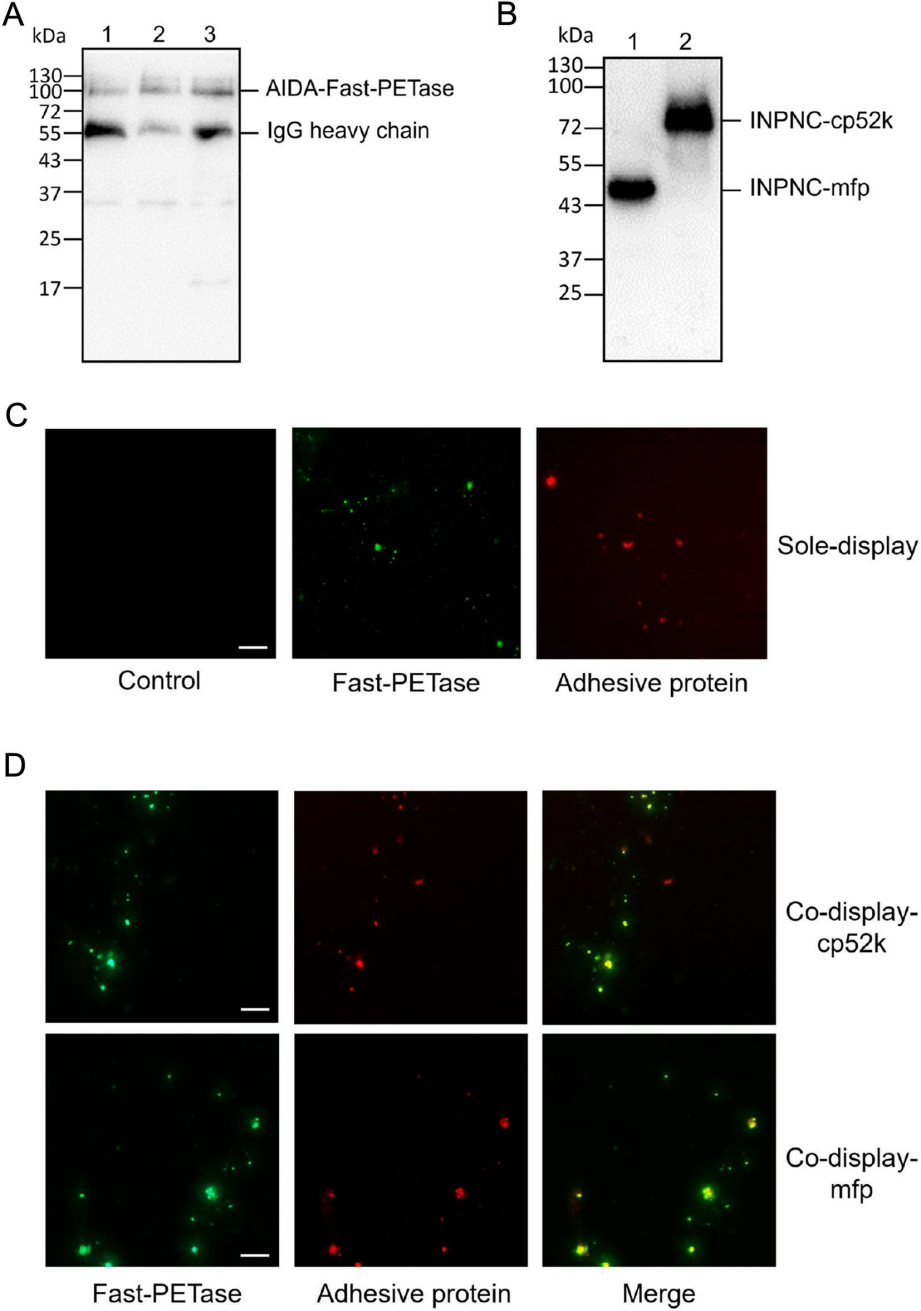
Western blots and fluorescent microscopy of immunostained *E. coli* cells. **A** Western blots of the immunoprecipitants from cell membrane fractions of *E. coli* co-display-cp52k (lane 1); *E. coli* co-display-mfp (lane 2) and *E. coli* sole-display (lane 3). The bands of mouse IgG heavy chain are indicated. **B** Western blots of cell membrane proteins for *E. coli* co-display-mfp (lane 1) and *E. coli* co-display-cp52k (lane 2). **C** Fluorescent microscopy of immunostained *E. coli* cells solely expressing Fast-PETase or adhesive protein on the surface and **D** *E. coli* cells simultaneously expressing Fast-PETase and adhesive protein on their surface. Scale bar = 10 μm

Subsequently, Fast-PETase (PETase^S121E/D186H/R224Q/N233K/R280A^, Lu et al. 2022) was displayed on the surfaces of *E. coli* cells through another anchor protein AIDA-I, an *E. coli* adhesin widely used for the display of recombinant proteins (Lattemann et al. 2000) (Fig. 3A, C), generating a sole-display strain. The displayed whole cells exhibited hydrolytic activity towards PET (Additional file 1: Fig. S1). Meanwhile, to compare display efficiency, different anchor proteins, including YeeJ (previously used to display IsPETase on the surfaces of *E. coli* UT56002, Gercke et al. 2021) and OmpA (another commonly-used anchor protein in Gram-negative bacteria, Long et al. 2021) were selected to display Fast-PETase on the surfaces. The comparison of PET degradation efficiency by three systems under the same conditions indicated that AIDA-I is the best anchor protein (Additional file 1: Fig. S2). Given the choice of more efficient PETase and anchor protein, it was reasonable to expect higher catalytic activity.

### Co-display of adhesive proteins and Fast-PETase on the surfaces of *E. coli* cells

After separately displaying adhesive proteins and Fast-PETase, we attempted to combine the adhesion module and catalytic module. To achieve coordinated expression of two fusion proteins without potential competition, the lac operator in the front of INPNC-cp52k and INPNC-mfp was deleted for constitutive expression of the adhesive proteins. These plasmids were co-transformed with the plasmid bearing AIDA-Fast-PETase into *E. coli* BL21(DE3) to generate two co-display strains (co-display-mfp and co-display-cp52k). The co-display strains were cultivated at 25℃ for cell growth and constitutive expression of adhesive proteins. Then, IPTG was added to induce the expression of AIDA-Fast-PETase with sequential expression of two fused adhesive proteins. Consequently, the expression of these fusion proteins was confirmed in the cell membrane fractions by Western blotting (Fig. 3A, B), and co-display of Fast-PETase and adhesive proteins on the bacterial surfaces was verified by immunofluorescence examination (Fig. 3D).

**Fig. 4:**
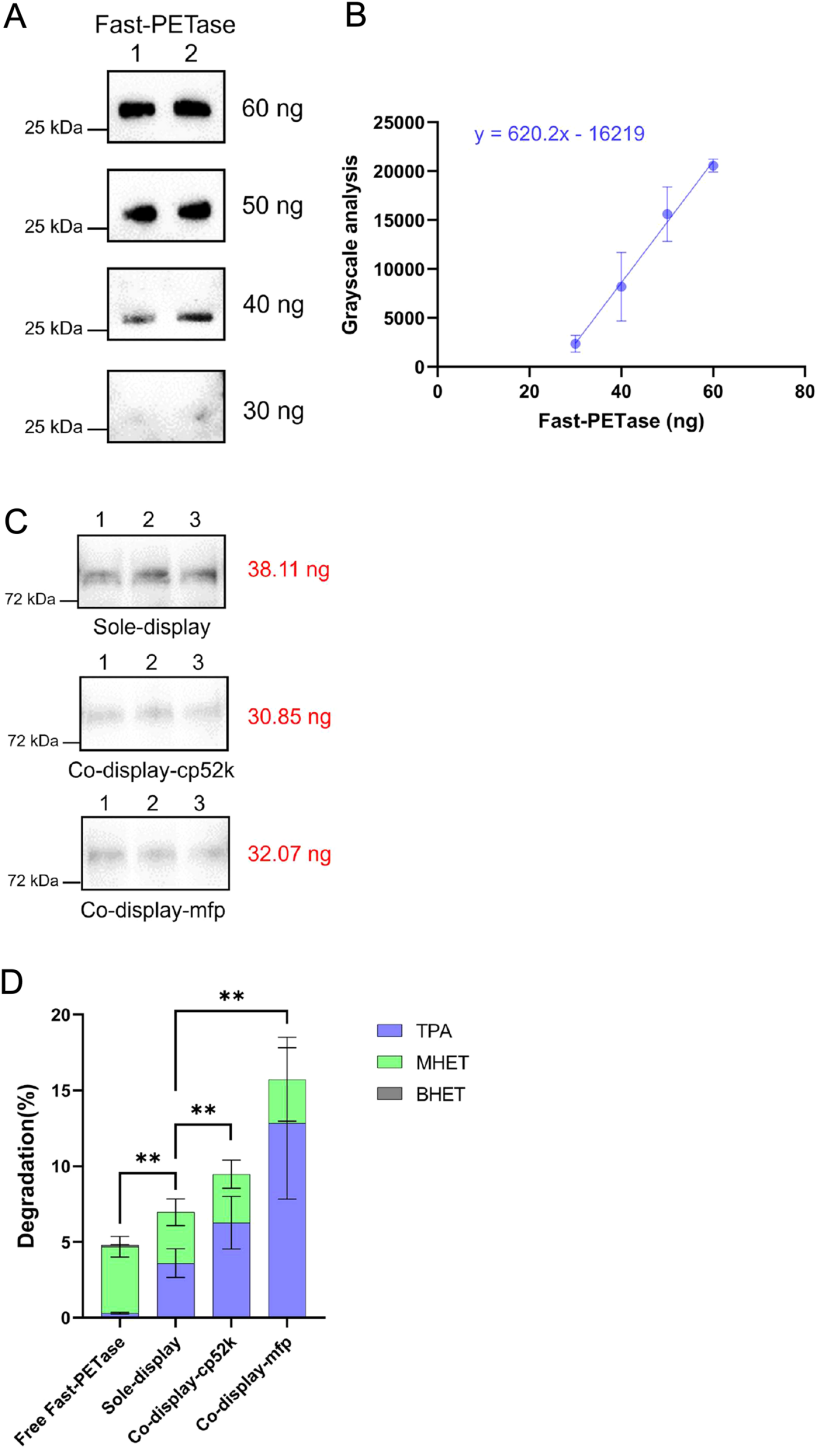
Quantification of Fast-PETase on the surface of engineered *E. coli* cells and PET degradation rates of engineered *E. coli* systems. (**A**) PETase-grayscale analysis of approximately 60 ng, 50 ng, 40 ng and 30 ng Fast-PETase by Western blotting. **B** Grayscale-protein concentration standard curve. The values represent the mean ± SD. **C** PETase-grayscale analysis of cell membrane proteins of *E. coli* strains with AIDA-PETase (sole-display), co-display-cp52k and co-display-mfp by Western blotting. Each lane contained cell membrane proteins from approximately 5.4 mg *E. coli* cells (wet weight). The samples of the same experiments were processed in parallel. **D** Comparison of PET degradation rate (%) for three *E. coli* cell systems and free Fast-PETase. All experiments were conducted in triplicate. The values are expressed as mean ± SD, and the asterisks denote the statistically significant difference (*p* < 0.01, unpaired t-test)

Next, Fast-PETase displayed on the surfaces of engineered strains was quantified by a grayscale-protein concentration standard curve (Fig. 4A–C). As a result, in the strain solely displaying Fast-PETase, 70 ng equivalent of Fast-PETase was displayed on the surfaces of every 8 mg wet weight of *E. coli* cells. For the co-display strains, 57 ng or 60 ng of Fast-PETase was displayed on the surfaces for *E. coli* co-display-cp52k or *E. coli* co-display-mfp, respectively. Together, the co-display of adhesive proteins with a PETase on the surfaces of E. coli cells was engineered and confirmed.

### PET biodegradation by engineered *E. coli* systems

To examine the efficiency and speed of our system, the rate of PET biodegradation was determined at 30 ℃ for 24 h with *E. coli* strains co-displaying adhesive proteins and Fast-PETase, *E. coli* strain solely displaying Fast-PETase and purified free Fast-PETase. Compared to solely displayed PETase on cell surfaces, both co-display-cp52k and co-display-mfp showed significant increase of PET degradation (Fig. 4D and Additional file 1: Fig. S1). The PET degradation rate of co-display-mfp in 24 h exceeded 15%, which was twofold more than that by solely displaying Fast-PETase. Compared to equal amount of free Fast-PETase, the degradation rate of sole-display, co-sdisplay-cp52k and co-display-mfp increased by 0.45, 1.39 and 2.31-fold, respectively (Table 1), demonstrating the advantages of co-display systems.

**Table 1:** Comparison of PET degradation efficiency by different biocatalysts

	Degradation (%)	Degradation (%) of equivalent free PETase	Fold improvement	TPA/Total products (%)
Free Fast-PETase^a^	4.81	**/**	**/**	6.33
Sole-display	6.96	4.81	0.45	51.72
Co-display-cp52k	9.47	3.96	1.39	66.20
Co-display-mfp	15.73	4.75	2.31	81.48

^a^ The amount of free Fast-PETase was equivalent to Fast-PETase displayed on the surface of *E. coli* AIDA-Fast-PETase

## Discussion

In this study, based on inherited advantages of *E. coli* cells, *E. coli* whole cell systems were constructed to simultaneously display marine organism-derived adhesive proteins and Fast-PETase on cell surfaces. The PET degradation efficiency of the co-display systems was significantly increased compared to *E. coli* cells that solely displayed Fast-PETase on the surfaces, proving the feasibility and utility of co-display approach with *E. coli* system. Because of the use of different PET materials and PETase enzymes for degradation tests (Chen et al. 2022; Gercke et al. 2021; Jia et al. 2022; Zhu et al. 2022), it was impossible for us to make a direct comparison with other reported systems. Nevertheless, over 15% degradation of amorphous PET in 24 h was the highest value to date. More importantly, the present study strongly suggested that simultaneous introduction of adhesive proteins and Fast-PETase to the surfaces of *E. coli* cells can effectively promote the process of PET biodegradation. Thus, the co-display systems in *E. coli* could be served as a convenient tool for expanding the treatment options for PET biodegradation.

According to previous reports (Yoshida et al. 2016; Joo et al. 2018), the hydrolytic process of PET by free PETase generally terminates at the point of MHET release. When we used free Fast-PETase for PET degradation, MHET accounted for the largest proportion (over 90%) of all degradation products (BHET, MHET and TPA) from PET, which is consistent with literatures that another MHETase should be involved in the hydrolysis of MHET (Yoshida et al. 2016; Knott et al. 2020). Differently, in our sole-display and co-display systems, TPA was the major product. Using co-display-mfp strain, over 80% of hydrolytic products was TPA, suggesting that our co-display systems could accomplish more thorough degradation of PET. This newly discovered difference on product profile could be due to favorable conformation of the enzyme and/or better contact with the substrate in our system, which requires further investigation to clarify. Collectively, the present study has provided a new treatment option for PET biodegradation, which may shed a light on practical applications.

### Supplementary Information

**Additional file 1: Figure S1.** HPLC chromatograms for the analysis of degradation products of PET and the standards of TPA, MHET and BHET. **Figure S2. **PET degradation rate (%) of three Fast-PETase-displaying strains of AIDA-Fast-PETase, YeeJ-Fast-PETase and OmpA-Fast-PETase. All experiments were conducted in triplicate. The values represent mean ± SD, and the asterisks denote statistically significant difference (p < 0.05, unpaired t-test). **Table S1**. Strains and plasmids used in this study. **Table S2.** Primers used in this study.

## Data Availability

The datasets used and/or analyzed during the current study are available from the corresponding author on reasonable request.

## Abbreviations

BHET: Bis(2-hydroxyethyl) terephthalate
CFU: Colony-forming unit
EG: Ethylene glycol
MHET: Mono(2-hydroxyethyl) terephthalate
PET: Polyethylene terephthalate
TPA: Terephthalic acid
