# Supplementary material for: Constructing Escherichia coli co-display systems for biodegradation of polyethylene terephthalate
Source: Bioresour Bioprocess. 2023 Dec 8;10(1):91. doi: 10.1186/s40643-023-00711-x (PMC10992762; doi:10.1186/s40643-023-00711-x)
Supplement: Supplementary file 1 — Additional file 1: Figure S1. HPLC chromatograms for the analysis of degradation products of PET and the standards of TPA, MHET and BHET. Figure S2. PET degradation rate (%) of three Fast-PETase-displaying strains of AIDA-Fast-PETase, YeeJ-Fast-PETase and OmpA-Fast-PETase. All experiments were conducted in triplicate. The values represent mean ± SD, and the asterisks denote statistically significant difference (p < 0.05, unpaired t-test). Table S1. Strains and plasmids used in this study. Table S2. Primers used in this study. [file 40643_2023_711_MOESM1_ESM.docx]

Additional material for

**Constructing *Escherichia coli* co-display systems for biodegradation of polyethylene terephthalate**

**Jiayu Hu and Yijun Chen^*^**

Laboratory of Chemical Biology and State Key Laboratory of Natural Medicines,

China Pharmaceutical University, Nanjing, Jiangsu 211198, P. R. China

*Correspondence: [yjchen@cpu.edu.cn](mailto:yjchen@cpu.edu.cn)

**
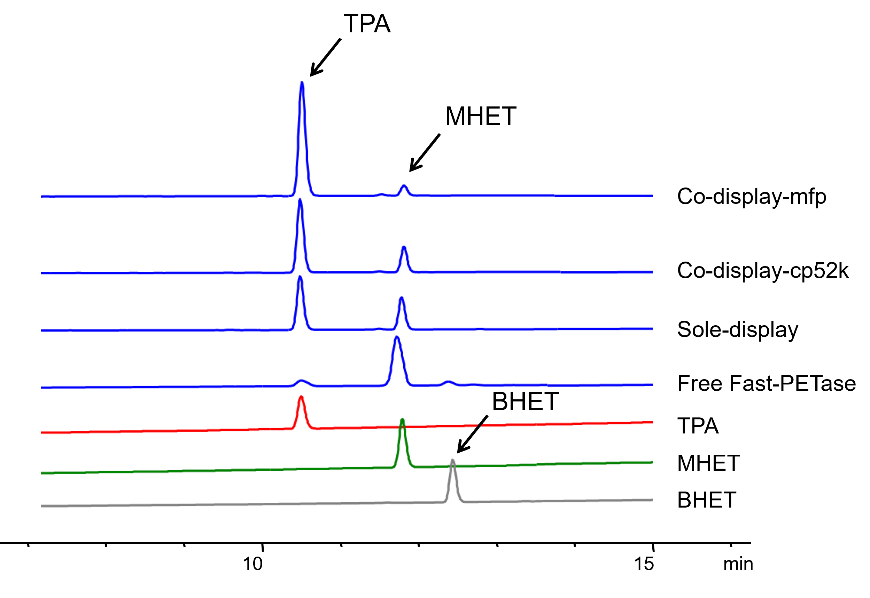
**

Additional **Figure 1** HPLC chromatograms for the analysis of degradation products of PET and the standards of TPA, MHET and BHET.

**
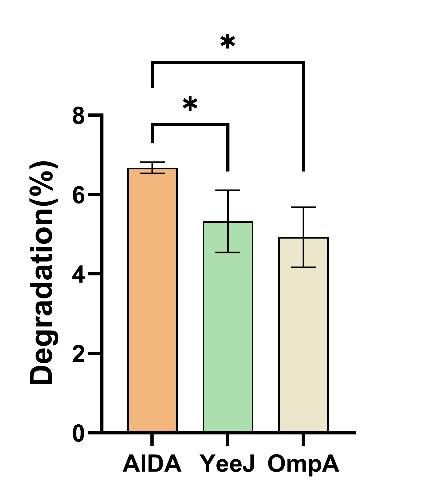
**

Additional **Figure 2** PET degradation rate (%) of three Fast-PETase-displaying strains of AIDA-Fast-PETase, YeeJ-Fast-PETase and OmpA-Fast-PETase. All experiments were conducted in triplicate. The values represent mean ± SD, and the asterisks denote statistically significant difference (p < 0.05, unpaired t-test).

Additional **Table 1**: Strains and plasmids used in this study

| Strains/plasmids | Description | Source |
| --- | --- | --- |
| **Plasmids** |  |  |
| pRSFDuet | The expression vector for cell surface display | Lab stock |
| pETDuet | The expression vector for cell surface display | Lab stock |
| pET22b | The expression vector for expression of Fast-PETase | Lab stock |
| pRSFDuet-mfp | INPNC and mfp-3 fusion protein; lac promoter | This study |
| pRSFDuet-cp52k | INPNC and cp52k fusion protein; lac promoter | This study |
| pRSFDuet-mfp-del | INPNC and mfp-3 fusion protein; lac promoter without lac operator | This study |
| pRSFDuet-cp52k-del | INPNC and cp52k fusion protein; lac promoter without lac operator | This study |
| pETDuet-AIDA-PETase | Fast-PETase and AIDA-I fusion protein; tac promoter | This study |
| pETDuet-YeeJ-PETase | Fast-PETase and YeeJ fusion protein; tac promoter | This study |
| pETDuet-OmpA-PETase | Fast-PETase and AIDA-I fusion protein; tac promoter | This study |
| pET22b-PETase | Fast-PETase with N-terminus His_6_ tag | This study |
| **Strains** |  |  |
| *E. coli* BL21(DE3) | F-, lon-11, Δ(ompT-nfrA)885, Δ(galM-ybhJ)884, DE3[lacI lacUV5-T7 gene 1 ind1 sam7 nin5], Δ46, [mal+]K-12(λS), hsdS10 | Lab stork |
| *E. coli* INPNC-mfp | *E. coli* BL21(DE3) harboring pRSFDuet-mfp | This study |
| *E. coli* INPNC-cp52k | *E. coli* BL21(DE3) harboring pRSFDuet-cp52k | This study |
| *E. coli* AIDA-Fast-PETase | *E. coli* BL21(DE3) harboring pETDuet-AIDA-PETase | This study |
| *E. coli* YeeJ-Fast-PETase | *E. coli* BL21(DE3) harboring pETDuet-YeeJ-PETase | This study |
| *E. coli* OmpA-Fast-PETase | *E. coli* BL21(DE3) harboring pETDuet-OmpA-PETase | This study |
| *E. coli* co-display-mfp | *E. coli* BL21(DE3) harboring pRSFDuet-mfp-del and pETDuet-PETase | This study |
| *E. coli* co-display-cp52k | *E. coli* BL21(DE3) harboring pRSFDuet-cp52k-del and pETDuet-PETase | This study |
| *E. coli* Fast-PETase | *E. coli* BL21(DE3) harboring pET22b-PETase | This study |

Additional **Table 2**: Primers used in this study

| Primers | Sequence |
| --- | --- |
| Lac-F | TGCGACTCCTGCATTAGGAGGCAGTGAGCGCAACG |
| Lac-R | TTTCTACAGGGGAATTGTTATCCGCTCACAATTCC |
| INP-F | TTAATAAGGAGATATACCATGGGCACCCTGGATAAAGCGCTGGTG |
| INP-R | GCCCGGATCTTTCACTTCAATC |
| Mfp-F | TGAAGTGAAAGATCCGGGCGCGGATTATTATGGCCCGAAATATGG |
| Myc-mfp-R | TTAAGCATTATGCGGCCGCAAGCTTTTAATAATATTTGCGGCCCCAGCGG |
| Myc-mfp-F | ATCTCCGAAGAGGACCTGTAAAAGCTTGCGGCCGC |
| Myc-cp52k-R | CAGGTCCTCTTCGGAGATCAGCTTCTGTTCTTTGCCCGCAAATTTCGGG |
| Myc-cp52k-F | ATCTCCGAAGAGGACCTGTAACTCGAGatcccaatggcAAG |
| Del-lacO-F | ACAATTCCCCTGTAGAAATAATTTTG |
| Del-lacO-R | TTATTTCTACAGGGGAATTGTccacacaacatacgagccg |
| VF | ATGCCCTCATTCAGCATTTGC |
| VR | AAATGCTGAATGAGGGCATCG |
| PETase-F | GTATAAGAAGGAGATATACATATGAAATACCTGCTGCCGACCGC |
| PETase-R | TGCGGCCGCAAGCTTG |
| PelB-F | ATACATATGAAATACCTGCTGCCG |
| PelB-R | ATATCCATGGCCATCGTCGG |
| AIDA-F | AGCAAGCTTGCGGCCGCAgggcaggatacttccgtcg |
| AIDA-R | CAGCGGTTTCTTTACCAGACTCGAGttaccatttcaccgtcatcgac |
| His-F | TGCCCAGCCGACGATGGCCCATCACCATCACCATCATGCGCAAACCAACCCATACG |
| His-R | GTATGGGTTGGTTTGCGCATGATGGTGATGGTGATGGGCCATCGTCGGCTGG |
| His-PETase-F | TATACATATGCATCACCATCACCATCATGCGCAAACCAACCCATACG |
| His-PETase-R | AACACTCGAGTCAGCTGCAGTTCGCGGTG |
| OmpA-F | GTATAAGAAGGAGATATACATATGAAGGCGACCAAACTGGTTC |
| OmpA-R | ATGGGTTGGTTTGCGCCATGCCCGGAATGCCATTATCC |
| YeeJ-F | GTATAAGAAGGAGATATACATATGggaattaaactacgccgcttaactg |
| YeeJ-R | ATGGGTTGGTTTGCGCCATatctttctggcttagcgtaggtg |
| PETase-F1 | ATGGCGCAAACCAACCC |
| PETase-R1 | TACAGGTCCTCTTCGGAGATCAGCTTCTGTTCGCTGCAGTTCGCGGTG |
| PETase-R2 | TTCTTTACCAGACTCGAGTTACAGGTCCTCTTCGGAGATC |
